# 1-Acetyl-5-meth­oxy-4-(phenyl­sulfan­yl)imidazolidin-2-one

**DOI:** 10.1107/S1600536814000117

**Published:** 2014-01-15

**Authors:** Joel T. Mague, Alaa A.-M. Abdel-Aziz, Adel S. El-Azab, Amer M. Alanazi

**Affiliations:** a Department of Chemistry, Tulane University, New Orleans, LA 70118, USA; b Department of Pharmaceutical Chemistry, College of Pharmacy, King Saud University, Riyadh 11451, Saudi Arabia; c Department of Medicinal Chemistry, Faculty of Pharmacy, University of Mansoura, Mansoura 35516, Egypt; d Department of Organic Chemistry, Faculty of Pharmacy, Al-Azhar University, Cairo 11884, Egypt

## Abstract

The title compound, C_12_H_14_N_2_O_3_S, crystallizes with two independent mol­ecules (*A* and *B*) in the asymmetric unit. The five-membered imidazolidin-2-one rings in both mol­ecules are twisted about the C—C bond. In the crystal, the *A* and *B* mol­ecules are associated *via* pairs of N—H⋯O hydrogen bonds, forming *A*–*B* dimers. These dimers are linked *via* C—H⋯S hydrogen bonds, forming double dimers, which are in turn linked *via* C—H⋯O hydrogen bonds forming two-dimensional networks lying parallel to (001). There are also C—H⋯π inter­actions present, which consolide the layers and link them, so forming a three-dimensional structure.

## Related literature   

For the anti­tumor activity of imidazolidinones, see: Abdel-Aziz *et al.* (2012 ▶); Lee *et al.* (2000 ▶); Kim *et al.* (2003 ▶). For related crystal structures, see: Park *et al.* (2000 ▶); Abdel-Aziz *et al.* (2012 ▶); Kapon & Reisner (1989 ▶). For ring conformation analysis, see: Cremer & Pople (1975 ▶).
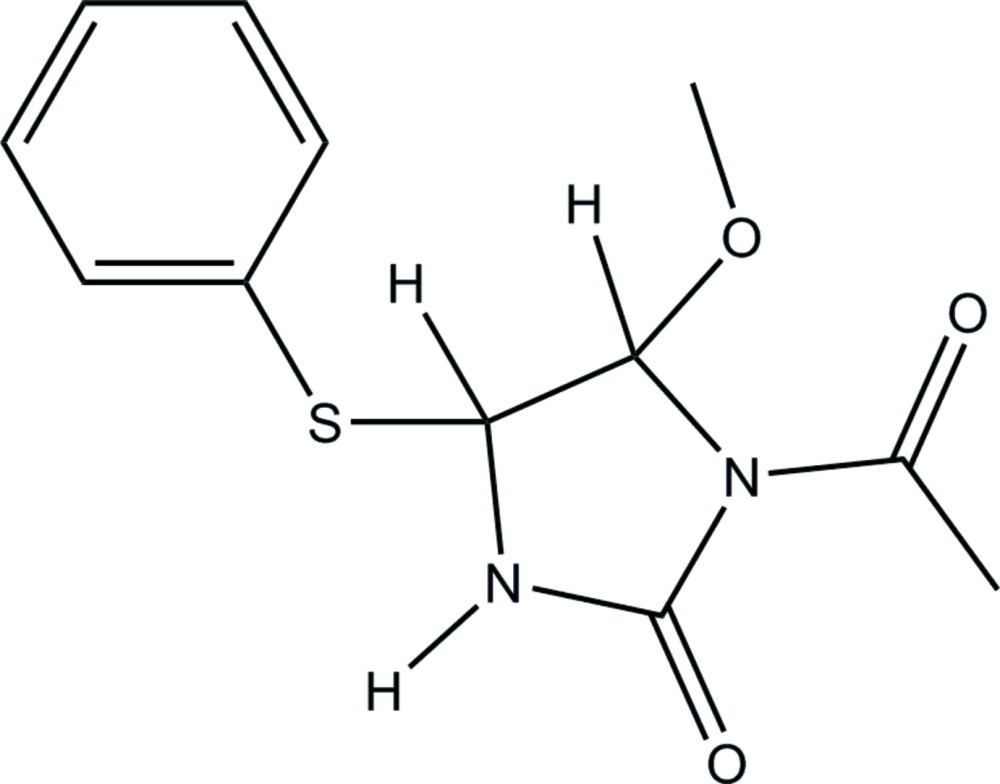


## Experimental   

### 

#### Crystal data  

C_12_H_14_N_2_O_3_S
*M* *_r_* = 266.31
Triclinic, 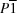

*a* = 9.0371 (8) Å
*b* = 12.0642 (10) Å
*c* = 12.8369 (11) Å
α = 93.066 (1)°
β = 105.869 (1)°
γ = 111.574 (1)°
*V* = 1233.20 (18) Å^3^
*Z* = 4
Mo *K*α radiation
μ = 0.26 mm^−1^
*T* = 100 K
0.25 × 0.14 × 0.10 mm

#### Data collection  

Bruker SMART APEX CCD diffractometer
Absorption correction: multi-scan (*SADABS*; Bruker, 2010 ▶) *T*_min_ = 0.847, *T*_max_ = 0.974
22009 measured reflections
6246 independent reflections
5490 reflections with *I* > 2σ(*I*)
*R*_int_ = 0.034

#### Refinement  

*R*[*F* ^2^ > 2σ(*F* ^2^)] = 0.034
*wR*(*F* ^2^) = 0.095
*S* = 1.03
6246 reflections
329 parameters
H-atom parameters constrained
Δρ_max_ = 0.48 e Å^−3^
Δρ_min_ = −0.35 e Å^−3^

### 

Data collection: *APEX2* (Bruker, 2010 ▶); cell refinement: *SAINT* (Bruker, 2010 ▶); data reduction: *SAINT*; program(s) used to solve structure: *SHELXS97* (Sheldrick, 2008 ▶); program(s) used to refine structure: *SHELXL97* (Sheldrick, 2008 ▶); molecular graphics: *DIAMOND* (Brandenburg & Putz, 2012 ▶); software used to prepare material for publication: *SHELXTL* (Sheldrick, 2008 ▶).

## Supplementary Material

Crystal structure: contains datablock(s) I, global. DOI: 10.1107/S1600536814000117/su2683sup1.cif

Structure factors: contains datablock(s) I. DOI: 10.1107/S1600536814000117/su2683Isup2.hkl

Supporting information file. DOI: 10.1107/S1600536814000117/su2683Isup3.cml

CCDC reference:

Additional supporting information:  crystallographic information; 3D view; checkCIF report

## Figures and Tables

**Table 1:** Hydrogen-bond geometry (Å, °) *Cg*1 and *Cg*2 are the centroids of rings C1–C6 and C13–C18, respectively.

*D*—H⋯*A*	*D*—H	H⋯*A*	*D*⋯*A*	*D*—H⋯*A*
N1—H1*N*⋯O6^i^	0.88	1.99	2.8483 (13)	165
N3—H3*N*⋯O3^ii^	0.89	1.99	2.8623 (13)	167
C8—H8⋯S2^iii^	1.00	2.86	3.7891 (12)	156
C7—H7⋯O3^iv^	1.00	2.63	3.4150 (15)	135
C18—H18⋯O3^ii^	0.95	2.55	3.4790 (15)	167
C20—H20⋯O1^v^	1.00	2.47	3.4604 (15)	173
C2—H2⋯*Cg*2^iii^	0.95	2.83	3.5830 (17)	137
C15—H15⋯*Cg*1	0.95	2.68	3.4496 (15)	138

Symmetry codes: (i) 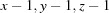; (ii) 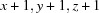; (iii) 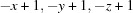; (iv) 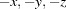; (v) 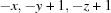.

**Fig. 1.**
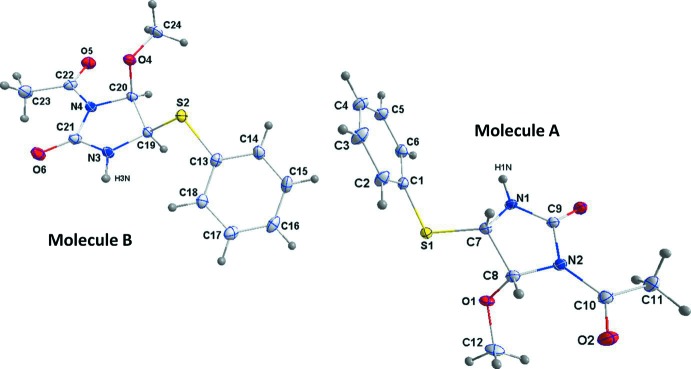
A view of the molecular structure of the two independent molecules (A and B) of the title compound, with atom labelling. The displacement ellipsoids are drawn at the 50% probability level.

**Fig. 2.**
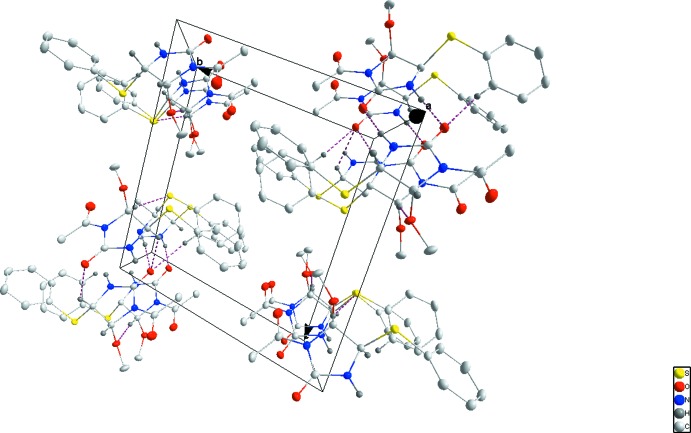
A view along the a axis of the crystal packing of the title compound. The hydrogen bonds are shown as dashed lines (see Table 1 for details).

## supplementary crystallographic information

### 1. Comment

Imidazolidinones are an interesting class of compounds possessing antitumor activity (Abdel-Aziz *et al.*, 2012; Lee *et al.*, 2000; Kim *et al.*, 2003). As a continuation of our studies on new biologically active compounds we report herein on the synthesis and crystal structure of the title compound.

The title compound crystallizes with two independent molecules (A and B) in the asymmetric unit, Fig. 1. The 5-membered rings (N1/C7/C8/N2/C9 and N3/C19/C20/N4/C21) are twisted about the C7—C8 and C19—C20 bonds, respectively, with puckering parameters (Cremer & Pople, 1975) of Q^2^ = 0.214 (1) Å; φ^2^ = 59.8 (3)° for molecule A and Q^2^ = 0.181 (1) Å; φ^2^ = 229.3 (4)° for molecule B.

In the crystal the A and B molecules are linked *via* pairs of N—H···O hydrogen bonds forming dimer-like arrangements (Table 1 and Fig. 2). The phenyl groups project outwards on both edges of the relatively flat central portion of the dimer. These dimers are linked via C—H···S hydrogen bonds forming double-dimers. These units are linked via C—H···O hydrogen bonds forming two-dimensional networks lying parallel to the ab plane. There are also C—H···π interactions present consolidating the layers and linking them so forming a three-dimensional structure - see Table 1 for details of the hydrogen bonding and C—H···π interactions.

### 2. Experimental

Trifluoroacetic acid (0.3 equiv) was added dropwise to a stirred solution of 1-acetyl-4,5-dimethoxyimidazolidin-2-one (1 equiv) and thiophenol (1 equiv) in dry CH_3_CN (0.01 mol/*L*) over a period of 15 min at room temperature. After being stirred for 2 h at room temperature, the mixture was quenched by adding aqueous ammonium chloride solution (5 ml), extracted with ethyl acetate, washed with brine and dried over anhydrous sodium sulfate. The product obtained after evaporation of the solvent was purified by column chromatography using mixture of hexane and CHCl_3_ as eluent. Colourless rod-like crystals were obtained by slow evaporation of the eluent solution.

### 3. Refinement

All H atoms were placed in idealized positions and allowed to ride on the respective parent atom: N—H = 0.88 & 0.89 Å, C—H = 0.95 - 1.00 Å with *U*_iso_(H) = 1.5*U*_eq_(C-methyl) and = 1.2*U*_eq_(N,C) for other H atoms.

### Crystal data

**Table:**

C_12_H_14_N_2_O_3_S	*Z* = 4
*M**_r_* = 266.31	*F*(000) = 560
Triclinic, *P*1	*D*_x_ = 1.434 Mg m^−^^3^
Hall symbol: -P 1	Mo *K*α radiation, λ = 0.71073 Å
*a* = 9.0371 (8) Å	Cell parameters from 9891 reflections
*b* = 12.0642 (10) Å	θ = 2.3–29.1°
*c* = 12.8369 (11) Å	µ = 0.26 mm^−^^1^
α = 93.066 (1)°	*T* = 100 K
β = 105.869 (1)°	Rod, colourless
γ = 111.574 (1)°	0.25 × 0.14 × 0.10 mm
*V* = 1233.20 (18) Å^3^	

### Data collection

**Table:**

Bruker SMART APEX CCD diffractometer	6246 independent reflections
Radiation source: fine-focus sealed tube	5490 reflections with *I* > 2σ(*I*)
Graphite monochromator	*R*_int_ = 0.034
φ and ω scans	θ_max_ = 29.1°, θ_min_ = 1.8°
Absorption correction: multi-scan (*SADABS*; Bruker, 2010)	*h* = −12→11
*T*_min_ = 0.847, *T*_max_ = 0.974	*k* = −16→16
22009 measured reflections	*l* = −17→17

### Refinement

**Table:**

Refinement on *F*^2^	Primary atom site location: structure-invariant direct methods
Least-squares matrix: full	Secondary atom site location: difference Fourier map
*R*[*F*^2^ > 2σ(*F*^2^)] = 0.034	Hydrogen site location: inferred from neighbouring sites
*wR*(*F*^2^) = 0.095	H-atom parameters constrained
*S* = 1.03	*w* = 1/[σ^2^(*F*_o_^2^) + (0.0516*P*)^2^ + 0.3898*P*] where *P* = (*F*_o_^2^ + 2*F*_c_^2^)/3
6246 reflections	(Δ/σ)_max_ = 0.001
329 parameters	Δρ_max_ = 0.48 e Å^−^^3^
0 restraints	Δρ_min_ = −0.35 e Å^−^^3^

### Special details

**Table:**

Geometry. All e.s.d.'s (except the e.s.d. in the dihedral angle between two l.s. planes) are estimated using the full covariance matrix. The cell e.s.d.'s are taken into account individually in the estimation of e.s.d.'s in distances, angles and torsion angles; correlations between e.s.d.'s in cell parameters are only used when they are defined by crystal symmetry. An approximate (isotropic) treatment of cell e.s.d.'s is used for estimating e.s.d.'s involving l.s. planes.
Refinement. Refinement of *F*^2^ against ALL reflections. The weighted *R*-factor *wR* and goodness of fit *S* are based on *F*^2^, conventional *R*-factors *R* are based on *F*, with *F* set to zero for negative *F*^2^. The threshold expression of *F*^2^ > σ(*F*^2^) is used only for calculating *R*-factors(gt) *etc*. and is not relevant to the choice of reflections for refinement. *R*-factors based on *F*^2^ are statistically about twice as large as those based on *F*, and *R*- factors based on ALL data will be even larger. H-atoms attached to carbon were placed in calculated positions (C—H = 0.95 - 0.98 Å) while those attached to nitrogen were placed in locations derived from a difference map. All were included as riding contributions with isotropic displacement parameters 1.2 - 1.5 times those of the attached atoms.

### Fractional atomic coordinates and isotropic or equivalent isotropic displacement parameters (Å^2^)

**Table:**

	*x*	*y*	*z*	*U*_iso_*/*U*_eq_	
S1	0.04876 (4)	0.16574 (3)	0.33282 (2)	0.01531 (8)	
O1	0.00144 (11)	−0.07345 (8)	0.32035 (7)	0.01676 (18)	
O2	0.08977 (13)	−0.24894 (9)	0.16722 (8)	0.0265 (2)	
O3	−0.25546 (11)	−0.12264 (8)	−0.01801 (7)	0.01828 (18)	
N1	−0.09455 (13)	0.04366 (9)	0.11887 (8)	0.0155 (2)	
H1N	−0.1380	0.0929	0.0891	0.019*	
N2	−0.03201 (12)	−0.11728 (9)	0.13027 (8)	0.0144 (2)	
C1	0.11335 (15)	0.31463 (10)	0.30058 (9)	0.0145 (2)	
C2	0.28336 (16)	0.38990 (11)	0.33308 (11)	0.0193 (2)	
H2	0.3646	0.3607	0.3696	0.023*	
C3	0.33403 (16)	0.50771 (12)	0.31202 (12)	0.0228 (3)	
H3	0.4501	0.5583	0.3330	0.027*	
C4	0.21611 (17)	0.55186 (12)	0.26060 (11)	0.0205 (3)	
H4	0.2510	0.6327	0.2472	0.025*	
C5	0.04669 (16)	0.47702 (12)	0.22883 (10)	0.0197 (2)	
H5	−0.0342	0.5070	0.1934	0.024*	
C6	−0.00562 (15)	0.35878 (11)	0.24838 (10)	0.0178 (2)	
H6	−0.1218	0.3081	0.2264	0.021*	
C7	0.05695 (14)	0.08366 (10)	0.21295 (9)	0.0131 (2)	
H7	0.1554	0.1347	0.1912	0.016*	
C8	0.07070 (14)	−0.03672 (10)	0.23658 (9)	0.0137 (2)	
H8	0.1896	−0.0288	0.2567	0.016*	
C9	−0.14064 (15)	−0.06988 (10)	0.06753 (9)	0.0137 (2)	
C10	−0.01687 (16)	−0.22561 (11)	0.10365 (10)	0.0172 (2)	
C11	−0.13220 (18)	−0.30806 (11)	−0.00263 (11)	0.0217 (3)	
H11A	−0.1031	−0.3779	−0.0115	0.033*	
H11B	−0.1205	−0.2641	−0.0639	0.033*	
H11C	−0.2483	−0.3360	−0.0019	0.033*	
C12	0.05831 (19)	−0.15414 (13)	0.38084 (11)	0.0250 (3)	
H12A	−0.0011	−0.2364	0.3385	0.037*	
H12B	0.0359	−0.1526	0.4513	0.037*	
H12C	0.1791	−0.1286	0.3942	0.037*	
S2	0.45632 (4)	0.91180 (3)	0.63984 (2)	0.01550 (8)	
O4	0.47788 (11)	1.14846 (8)	0.66767 (7)	0.01680 (18)	
O5	0.38711 (12)	1.30406 (8)	0.81309 (8)	0.0219 (2)	
O6	0.70722 (12)	1.16581 (8)	1.01055 (7)	0.02057 (19)	
N3	0.57467 (13)	1.01168 (9)	0.86090 (8)	0.0165 (2)	
H3N	0.6113	0.9619	0.8987	0.020*	
N4	0.50197 (13)	1.16831 (9)	0.85376 (8)	0.0147 (2)	
C13	0.38049 (15)	0.75462 (11)	0.64573 (10)	0.0156 (2)	
C14	0.27394 (16)	0.67501 (12)	0.54824 (10)	0.0209 (3)	
H14	0.2400	0.7059	0.4835	0.025*	
C15	0.21730 (17)	0.55073 (12)	0.54547 (11)	0.0231 (3)	
H15	0.1459	0.4971	0.4786	0.028*	
C16	0.26439 (17)	0.50477 (12)	0.63972 (12)	0.0226 (3)	
H16	0.2245	0.4198	0.6379	0.027*	
C17	0.37027 (18)	0.58348 (12)	0.73695 (11)	0.0216 (3)	
H17	0.4022	0.5520	0.8017	0.026*	
C18	0.42995 (16)	0.70802 (11)	0.74040 (10)	0.0185 (2)	
H18	0.5041	0.7613	0.8069	0.022*	
C19	0.43039 (15)	0.97353 (10)	0.76257 (9)	0.0134 (2)	
H19	0.3302	0.9139	0.7771	0.016*	
C20	0.40610 (14)	1.09291 (10)	0.74557 (9)	0.0139 (2)	
H20	0.2851	1.0794	0.7258	0.017*	
C21	0.60722 (15)	1.11992 (11)	0.91856 (9)	0.0149 (2)	
C22	0.49203 (16)	1.27940 (11)	0.87707 (10)	0.0164 (2)	
C23	0.61395 (18)	1.36279 (12)	0.98098 (11)	0.0233 (3)	
H23A	0.5917	1.4359	0.9886	0.035*	
H23B	0.7285	1.3850	0.9784	0.035*	
H23C	0.6014	1.3220	1.0440	0.035*	
C24	0.35893 (17)	1.16093 (12)	0.57410 (10)	0.0222 (3)	
H24A	0.2766	1.0806	0.5347	0.033*	
H24B	0.4175	1.2038	0.5250	0.033*	
H24C	0.3014	1.2069	0.5986	0.033*	

### Atomic displacement parameters (Å^2^)

**Table:**

	*U*^11^	*U*^22^	*U*^33^	*U*^12^	*U*^13^	*U*^23^
S1	0.01895 (15)	0.01327 (14)	0.01343 (13)	0.00573 (11)	0.00561 (11)	0.00232 (10)
O1	0.0190 (4)	0.0173 (4)	0.0165 (4)	0.0087 (3)	0.0062 (3)	0.0084 (3)
O2	0.0328 (5)	0.0231 (5)	0.0284 (5)	0.0198 (4)	0.0046 (4)	0.0058 (4)
O3	0.0205 (4)	0.0160 (4)	0.0159 (4)	0.0085 (4)	0.0008 (3)	0.0003 (3)
N1	0.0187 (5)	0.0136 (5)	0.0135 (4)	0.0094 (4)	0.0001 (4)	0.0020 (4)
N2	0.0154 (5)	0.0123 (5)	0.0154 (4)	0.0070 (4)	0.0028 (4)	0.0022 (4)
C1	0.0172 (5)	0.0122 (5)	0.0143 (5)	0.0061 (4)	0.0055 (4)	0.0009 (4)
C2	0.0166 (6)	0.0163 (6)	0.0263 (6)	0.0088 (5)	0.0058 (5)	0.0028 (5)
C3	0.0165 (6)	0.0156 (6)	0.0360 (7)	0.0056 (5)	0.0089 (5)	0.0042 (5)
C4	0.0245 (6)	0.0155 (6)	0.0268 (6)	0.0101 (5)	0.0124 (5)	0.0061 (5)
C5	0.0212 (6)	0.0193 (6)	0.0218 (6)	0.0117 (5)	0.0064 (5)	0.0049 (5)
C6	0.0158 (5)	0.0181 (6)	0.0181 (5)	0.0065 (5)	0.0041 (4)	0.0019 (5)
C7	0.0143 (5)	0.0122 (5)	0.0132 (5)	0.0059 (4)	0.0039 (4)	0.0029 (4)
C8	0.0132 (5)	0.0132 (5)	0.0146 (5)	0.0058 (4)	0.0032 (4)	0.0035 (4)
C9	0.0155 (5)	0.0140 (5)	0.0137 (5)	0.0076 (4)	0.0053 (4)	0.0042 (4)
C10	0.0212 (6)	0.0141 (5)	0.0201 (6)	0.0089 (5)	0.0094 (5)	0.0051 (5)
C11	0.0295 (7)	0.0140 (6)	0.0223 (6)	0.0101 (5)	0.0077 (5)	0.0008 (5)
C12	0.0337 (7)	0.0231 (7)	0.0226 (6)	0.0147 (6)	0.0092 (6)	0.0140 (5)
S2	0.01739 (15)	0.01489 (15)	0.01454 (14)	0.00679 (11)	0.00530 (11)	0.00111 (11)
O4	0.0169 (4)	0.0185 (4)	0.0150 (4)	0.0068 (3)	0.0048 (3)	0.0056 (3)
O5	0.0236 (5)	0.0193 (4)	0.0261 (5)	0.0132 (4)	0.0063 (4)	0.0056 (4)
O6	0.0254 (5)	0.0183 (4)	0.0159 (4)	0.0120 (4)	−0.0006 (4)	−0.0010 (3)
N3	0.0202 (5)	0.0145 (5)	0.0140 (4)	0.0103 (4)	0.0000 (4)	0.0007 (4)
N4	0.0166 (5)	0.0139 (5)	0.0140 (4)	0.0079 (4)	0.0033 (4)	0.0015 (4)
C13	0.0144 (5)	0.0149 (5)	0.0190 (5)	0.0072 (4)	0.0062 (4)	0.0006 (4)
C14	0.0215 (6)	0.0206 (6)	0.0190 (6)	0.0104 (5)	0.0022 (5)	−0.0012 (5)
C15	0.0191 (6)	0.0187 (6)	0.0265 (6)	0.0064 (5)	0.0030 (5)	−0.0061 (5)
C16	0.0226 (6)	0.0146 (6)	0.0332 (7)	0.0068 (5)	0.0142 (5)	0.0002 (5)
C17	0.0278 (7)	0.0205 (6)	0.0225 (6)	0.0126 (5)	0.0126 (5)	0.0051 (5)
C18	0.0204 (6)	0.0177 (6)	0.0174 (5)	0.0076 (5)	0.0062 (5)	0.0002 (5)
C19	0.0143 (5)	0.0128 (5)	0.0132 (5)	0.0061 (4)	0.0038 (4)	0.0014 (4)
C20	0.0139 (5)	0.0137 (5)	0.0139 (5)	0.0058 (4)	0.0038 (4)	0.0023 (4)
C21	0.0170 (5)	0.0144 (5)	0.0149 (5)	0.0077 (5)	0.0050 (4)	0.0029 (4)
C22	0.0194 (6)	0.0138 (5)	0.0188 (5)	0.0072 (5)	0.0090 (5)	0.0040 (4)
C23	0.0322 (7)	0.0152 (6)	0.0211 (6)	0.0114 (5)	0.0042 (5)	−0.0001 (5)
C24	0.0257 (7)	0.0235 (6)	0.0161 (5)	0.0112 (5)	0.0021 (5)	0.0063 (5)

### Geometric parameters (Å, º)

**Table:**

S1—C1	1.7830 (12)	S2—C13	1.7775 (13)
S1—C7	1.8186 (12)	S2—C19	1.8157 (12)
O1—C8	1.3998 (14)	O4—C20	1.4059 (14)
O1—C12	1.4301 (15)	O4—C24	1.4329 (14)
O2—C10	1.2115 (15)	O5—C22	1.2124 (15)
O3—C9	1.2264 (14)	O6—C21	1.2232 (15)
N1—C9	1.3483 (15)	N3—C21	1.3545 (15)
N1—C7	1.4538 (14)	N3—C19	1.4496 (14)
N1—H1N	0.8764	N3—H3N	0.8921
N2—C10	1.3975 (15)	N4—C22	1.3980 (15)
N2—C9	1.4032 (14)	N4—C21	1.4017 (15)
N2—C8	1.4733 (15)	N4—C20	1.4609 (15)
C1—C2	1.3936 (17)	C13—C14	1.3957 (17)
C1—C6	1.3978 (17)	C13—C18	1.3977 (17)
C2—C3	1.3903 (18)	C14—C15	1.3897 (18)
C2—H2	0.9500	C14—H14	0.9500
C3—C4	1.3875 (18)	C15—C16	1.384 (2)
C3—H3	0.9500	C15—H15	0.9500
C4—C5	1.3886 (18)	C16—C17	1.3882 (19)
C4—H4	0.9500	C16—H16	0.9500
C5—C6	1.3887 (18)	C17—C18	1.3907 (18)
C5—H5	0.9500	C17—H17	0.9500
C6—H6	0.9500	C18—H18	0.9500
C7—C8	1.5413 (16)	C19—C20	1.5524 (16)
C7—H7	1.0000	C19—H19	1.0000
C8—H8	1.0000	C20—H20	1.0000
C10—C11	1.5006 (18)	C22—C23	1.4976 (17)
C11—H11A	0.9800	C23—H23A	0.9800
C11—H11B	0.9800	C23—H23B	0.9800
C11—H11C	0.9800	C23—H23C	0.9800
C12—H12A	0.9800	C24—H24A	0.9800
C12—H12B	0.9800	C24—H24B	0.9800
C12—H12C	0.9800	C24—H24C	0.9800
			
C1—S1—C7	99.86 (5)	C13—S2—C19	101.64 (6)
C8—O1—C12	115.46 (10)	C20—O4—C24	113.52 (9)
C9—N1—C7	113.25 (10)	C21—N3—C19	113.13 (10)
C9—N1—H1N	122.1	C21—N3—H3N	116.4
C7—N1—H1N	123.2	C19—N3—H3N	125.0
C10—N2—C9	128.26 (10)	C22—N4—C21	128.30 (10)
C10—N2—C8	121.10 (10)	C22—N4—C20	119.61 (10)
C9—N2—C8	110.62 (9)	C21—N4—C20	111.81 (9)
C2—C1—C6	119.69 (11)	C14—C13—C18	119.31 (11)
C2—C1—S1	119.75 (9)	C14—C13—S2	117.24 (10)
C6—C1—S1	120.47 (9)	C18—C13—S2	123.35 (9)
C3—C2—C1	119.98 (11)	C15—C14—C13	120.31 (12)
C3—C2—H2	120.0	C15—C14—H14	119.8
C1—C2—H2	120.0	C13—C14—H14	119.8
C4—C3—C2	120.41 (12)	C16—C15—C14	120.29 (12)
C4—C3—H3	119.8	C16—C15—H15	119.9
C2—C3—H3	119.8	C14—C15—H15	119.9
C3—C4—C5	119.56 (12)	C15—C16—C17	119.66 (12)
C3—C4—H4	120.2	C15—C16—H16	120.2
C5—C4—H4	120.2	C17—C16—H16	120.2
C4—C5—C6	120.64 (12)	C16—C17—C18	120.61 (12)
C4—C5—H5	119.7	C16—C17—H17	119.7
C6—C5—H5	119.7	C18—C17—H17	119.7
C5—C6—C1	119.71 (11)	C17—C18—C13	119.81 (11)
C5—C6—H6	120.1	C17—C18—H18	120.1
C1—C6—H6	120.1	C13—C18—H18	120.1
N1—C7—C8	102.27 (9)	N3—C19—C20	102.59 (9)
N1—C7—S1	114.45 (8)	N3—C19—S2	115.63 (8)
C8—C7—S1	111.08 (8)	C20—C19—S2	109.31 (8)
N1—C7—H7	109.6	N3—C19—H19	109.7
C8—C7—H7	109.6	C20—C19—H19	109.7
S1—C7—H7	109.6	S2—C19—H19	109.7
O1—C8—N2	111.86 (9)	O4—C20—N4	108.69 (9)
O1—C8—C7	108.49 (9)	O4—C20—C19	111.93 (9)
N2—C8—C7	101.79 (9)	N4—C20—C19	101.96 (9)
O1—C8—H8	111.4	O4—C20—H20	111.3
N2—C8—H8	111.4	N4—C20—H20	111.3
C7—C8—H8	111.4	C19—C20—H20	111.3
O3—C9—N1	126.89 (11)	O6—C21—N3	126.56 (11)
O3—C9—N2	125.95 (11)	O6—C21—N4	126.45 (11)
N1—C9—N2	107.15 (10)	N3—C21—N4	106.99 (10)
O2—C10—N2	118.56 (11)	O5—C22—N4	119.20 (11)
O2—C10—C11	122.56 (11)	O5—C22—C23	123.21 (11)
N2—C10—C11	118.88 (11)	N4—C22—C23	117.59 (11)
C10—C11—H11A	109.5	C22—C23—H23A	109.5
C10—C11—H11B	109.5	C22—C23—H23B	109.5
H11A—C11—H11B	109.5	H23A—C23—H23B	109.5
C10—C11—H11C	109.5	C22—C23—H23C	109.5
H11A—C11—H11C	109.5	H23A—C23—H23C	109.5
H11B—C11—H11C	109.5	H23B—C23—H23C	109.5
O1—C12—H12A	109.5	O4—C24—H24A	109.5
O1—C12—H12B	109.5	O4—C24—H24B	109.5
H12A—C12—H12B	109.5	H24A—C24—H24B	109.5
O1—C12—H12C	109.5	O4—C24—H24C	109.5
H12A—C12—H12C	109.5	H24A—C24—H24C	109.5
H12B—C12—H12C	109.5	H24B—C24—H24C	109.5
			
C7—S1—C1—C2	−88.48 (10)	C19—S2—C13—C14	135.75 (10)
C7—S1—C1—C6	95.09 (10)	C19—S2—C13—C18	−48.11 (12)
C6—C1—C2—C3	−0.98 (19)	C18—C13—C14—C15	0.18 (19)
S1—C1—C2—C3	−177.44 (10)	S2—C13—C14—C15	176.47 (10)
C1—C2—C3—C4	1.2 (2)	C13—C14—C15—C16	0.8 (2)
C2—C3—C4—C5	−0.9 (2)	C14—C15—C16—C17	−0.8 (2)
C3—C4—C5—C6	0.22 (19)	C15—C16—C17—C18	−0.3 (2)
C4—C5—C6—C1	0.03 (19)	C16—C17—C18—C13	1.24 (19)
C2—C1—C6—C5	0.35 (18)	C14—C13—C18—C17	−1.19 (19)
S1—C1—C6—C5	176.78 (9)	S2—C13—C18—C17	−177.25 (10)
C9—N1—C7—C8	−17.42 (13)	C21—N3—C19—C20	17.05 (13)
C9—N1—C7—S1	−137.63 (9)	C21—N3—C19—S2	135.93 (9)
C1—S1—C7—N1	−85.84 (9)	C13—S2—C19—N3	92.14 (9)
C1—S1—C7—C8	158.98 (8)	C13—S2—C19—C20	−152.75 (8)
C12—O1—C8—N2	91.43 (12)	C24—O4—C20—N4	−128.97 (10)
C12—O1—C8—C7	−157.09 (10)	C24—O4—C20—C19	119.19 (11)
C10—N2—C8—O1	−82.90 (13)	C22—N4—C20—O4	70.60 (13)
C9—N2—C8—O1	95.95 (11)	C21—N4—C20—O4	−103.86 (11)
C10—N2—C8—C7	161.46 (10)	C22—N4—C20—C19	−171.06 (10)
C9—N2—C8—C7	−19.68 (12)	C21—N4—C20—C19	14.49 (12)
N1—C7—C8—O1	−97.00 (10)	N3—C19—C20—O4	98.13 (10)
S1—C7—C8—O1	25.53 (11)	S2—C19—C20—O4	−25.09 (12)
N1—C7—C8—N2	21.08 (11)	N3—C19—C20—N4	−17.87 (11)
S1—C7—C8—N2	143.61 (8)	S2—C19—C20—N4	−141.09 (8)
C7—N1—C9—O3	−174.34 (12)	C19—N3—C21—O6	170.27 (12)
C7—N1—C9—N2	5.55 (13)	C19—N3—C21—N4	−8.52 (14)
C10—N2—C9—O3	8.5 (2)	C22—N4—C21—O6	2.6 (2)
C8—N2—C9—O3	−170.27 (11)	C20—N4—C21—O6	176.49 (12)
C10—N2—C9—N1	−171.40 (11)	C22—N4—C21—N3	−178.58 (12)
C8—N2—C9—N1	9.84 (13)	C20—N4—C21—N3	−4.72 (13)
C9—N2—C10—O2	177.09 (12)	C21—N4—C22—O5	−176.52 (12)
C8—N2—C10—O2	−4.27 (18)	C20—N4—C22—O5	10.04 (17)
C9—N2—C10—C11	−2.51 (19)	C21—N4—C22—C23	3.76 (19)
C8—N2—C10—C11	176.13 (11)	C20—N4—C22—C23	−169.67 (11)

### Hydrogen-bond geometry (Å, º)

Cg1 and Cg2 are the centroids of rings C1–C6 and C13–C18, respectively.

**Table:**

*D*—H···*A*	*D*—H	H···*A*	*D*···*A*	*D*—H···*A*
N1—H1*N*···O6^i^	0.88	1.99	2.8483 (13)	165
N3—H3*N*···O3^ii^	0.89	1.99	2.8623 (13)	167
C8—H8···S2^iii^	1.00	2.86	3.7891 (12)	156
C7—H7···O3^iv^	1.00	2.63	3.4150 (15)	135
C18—H18···O3^ii^	0.95	2.55	3.4790 (15)	167
C20—H20···O1^v^	1.00	2.47	3.4604 (15)	173
C2—H2···*Cg*2^iii^	0.95	2.83	3.5830 (17)	137
C15—H15···*Cg*1	0.95	2.68	3.4496 (15)	138

Symmetry codes: (i) *x*−1, *y*−1, *z*−1; (ii) *x*+1, *y*+1, *z*+1; (iii) −*x*+1, −*y*+1, −*z*+1; (iv) −*x*, −*y*, −*z*; (v) −*x*, −*y*+1, −*z*+1.
